# Exploring the Role of Krebs von den Lungen-6 in Severe to Critical COVID-19 Patients

**DOI:** 10.3390/life12081141

**Published:** 2022-07-28

**Authors:** Vito D’Agnano, Filippo Scialò, Francesco Perna, Lidia Atripaldi, Stefano Sanduzzi, Valentino Allocca, Maria Vitale, Lucio Pastore, Andrea Bianco, Fabio Perrotta

**Affiliations:** 1 Department of Translational Medical Sciences, University of Campania “L. Vanvitelli”, 80131 Naples, Italy; vito.dagnano@studenti.unicampania.it (V.D.); filippo.scialo@unicampania.it (F.S.); lidia.atripaldi@studenti.unicampania.it (L.A.); stefano.sanduzzi@studenti.unicampania.it (S.S.); valentino.allocca@unicampania.it (V.A.); andrea.bianco@unicampania.it (A.B.); 2 CEINGE-Biotecnologie Avanzate, 80131 Naples, Italy; vitalema@ceinge.unina.it (M.V.); lucio.pastore@unina.it (L.P.); 3 Section of Respiratory Diseases, Department of Clinical Medicine and Surgery, Monaldi Hospital, University of Naples Federico II, 80131 Naples, Italy; francesco.perna@unina.it; 4 Section of Biochemical Clinical Laboratory, Department of Clinical Medicine and Surgery, University of Naples Federico II, 80131 Naples, Italy; 5 Dipartimento di Medicina Molecolare e Biotecnologie Mediche, Università di Napoli Federico II, 80131 Naples, Italy

**Keywords:** COVID-19, SARS-CoV-2, Krebs von den Lungen-6, lung ultrasound score

## Abstract

COVID-19 encompasses a broad spectrum of clinical conditions caused by SARS-CoV-2 infection. More severe cases experience acute respiratory and/or multiorgan failure. KL-6 is a glycoprotein expressed mainly from type II alveolar cells with pro-fibrotic properties. Serum KL-6 concentrations have been found in patients with COVID-19. However, the relevance of KL-6 in patients with severe and critical COVID-19 has not been fully elucidated. Methods: Retrospective data from consecutive severe to critical COVID-19 patients were collected at UOC Clinica Pnuemologica “Vanvitelli”, A.O. dei Colli, Naples, Italy. The study included patients with a positive rhinopharyngeal swab for SARS-CoV-2 RNA with severe or critical COVID-19. Results: Among 87 patients, 24 had poor outcomes. The median KL-6 value in survivors was significantly lower when compared with dead or intubated patients (530 U/mL versus 1069 U/mL *p* < 0.001). KL-6 was correlated with body mass index (BMI) (r: 0.279, *p*: 0.009), lung ultrasound score (LUS) (r: 0.429, *p* < 0.001), Chung Score (r: 0.390, *p* < 0.001). KL-6 was associated with the risk of death or oro-tracheal intubation (IOT) after adjusting for gender, BMI, Charlson Index, Chung Score, and PaO_2_/FIO_2_ (OR 1.003 95% CI 1.001–1.004, *p* < 0.001). Serum KL-6 value of 968 has a sensitivity of 79.2%, specificity of 87.1%, PPV 70.4%, NPV 91.5%, AUC: O.85 for risk of death or IOT. Conclusions: The presented research highlights the relevance of serum KL-6 in severe to critical COVID-19 patients in predicting the risk of death or IOT.

## 1. Introduction

The COVID-19 syndrome caused by the new coronavirus SARS-CoV-2 is characterized by a broad range of clinical manifestations with different degrees of severity ranging from mild, moderate, severe, and critical. In the most severe and critical cases, symptoms include respiratory distress syndrome, a hyperinflammatory status caused by the cytokine storm syndrome, and a hypercoagulable state leading to disseminated intravascular coagulation (DIC), multiorgan failure, and death [1,2,3]. Therefore, since the beginning of this pandemic, many efforts have been adopted to find reliable biomarkers that in these patients could predict the need for intubation or mortality. Many studies have already shown that an increasing trend of biochemical parameters, such as CRP, IL-6, D-dimer, neutrophil, and lymphocyte count [4,5] gives only a limited picture of a patient’s clinical condition and the search for new biomarkers that positively correlate with the need for intubation and mortality is still of great importance. For this reason, in our study, we sought to characterize the role of Krebs von den Lungen-6 (KL-6) protein in a subset of patients admitted to our hospital that progressed to severe conditions. We aimed to understand if a correlation would exist between its level and lung function in severe and critical COVID-19 patients and therefore be used to predict the need for intubation and if it would correlate with mortality.

KL-6 is produced by the shedding of the extracellular domain of MUCIN 1, a glycoprotein mainly expressed by damaged alveolar type II cells and has been shown to have an anti-inflammatory action through the inhibition of Toll-like receptor signaling but also participates in lung cancer progression and development of fibrotic processes [6]. KL-6 is formed by a stretch of 20 amino acids rich in glycosylated serine and threonine residue forming a rigid structure that protrudes into the extracellular space and provides a barrier against microbial and virus attacks [6]. Interestingly, the shedding of MUCIN 1 and release of KL-6 in the serum is operated by ADAM17 [7], which is also responsible for the cleavage of ACE2, the main receptor of SARS-CoV-2 [8,9]. KL-6 has initially been identified as a tumor marker [10] but has successively been found to play a role in inducing fibroblast migration [11] and inhibiting cell–cell adhesion [12], and could be used as a marker of interstitial lung fibrosis (ILF) [13]. Few studies have begun to describe an increase in KL-6 level in COVID-19 patients [14,15,16,17,18,19] although its physiological role in the context of SARS-CoV-2 infection needs further elucidation. As discussed previously, to characterize this biomarker further, here we add to the existing evidence the study of KL-6 specifically in the context of COVID-19 patients with more severe phases of respiratory involvement. Here, to the best of our knowledge, we clearly describe for the first time a strong correlation between KL-6 levels and lung function assessed by LUS. Moreover, based on our data, we suggest that KL-6 could be used as a marker to predict the need for intubation, and it strongly correlates with mortality.

## 2. Materials and Methods

A total of 87 consecutive patients admitted to U.O.C. Clinica Pnuemologica “Vanvitelli”, A.O. dei Colli, Naples, Italy, have been included in the analysis. Demographic, clinical, laboratory, and treatment data were extracted from electronic medical records. To be included in the study, patients were required to have a positive rhinopharyngeal swab for SARS-CoV-2 RNA. Criteria for severe COVID-19 were clinical signs of pneumonia (fever, cough, dyspnoea) plus one of the following: respiratory rate > 30 breaths/min; severe respiratory distress; or SpO_2_ < 90% on room air [20]. Criteria for critical COVID-19 were (1) respiratory symptoms onset within 1 week; (2) chest imaging showing bilateral opacities, not fully explained by volume overload, and (3) PaO_2_/FIO_2_ < 300 mmHg with PEEP or CPAP > 5 cm H_2_O. [20] Peripheral blood samples were centrifuged at 500× *g* for 10 min. Chemiluminescence enzyme immunoassay (CLEIA) using a KL-6 antibody kit (LUMIPULSE G1200, Fujirebio, Tokyo, Japan), in line with the manufacturer’s protocol, has been employed to assess serum KL-6. The kit had a detection range between 50–10,000 U/mL, where the level in healthy donors has been specified to be between 118–627 U/mL.

The patients were followed up until discharge or in-hospital death. The respiratory support needed during hospitalization included a venturi mask (VM) or non-rebreathing mask (NRM), high-flow nasal cannula (HFNC), continuous positive airway pressure (CPAP), and/or pressure support non-invasive ventilation (NIV). Acute respiratory failure management was provided according to international recommendations [21]. PaO_2_/FIO_2_ was calculated based on the ratio between arterial O_2_ pressure and oxygen inspiratory fraction administered.

LUS was performed as the patient entered the ward according to the 12 field 12-region model, 6 on each side as otherwise reported. Artifacts have been categorized as follows: A-line, horizontal artifacts observed in normal lungs; B-lines: vertical artifacts in a variety of patterns including focal and confluent; consolidations, including small peripheral subpleural or multifocal, translobar consolidation with occasional mobile air bronchograms and white lung. Finally, the state of pleural line was assessed. The findings were classified according to the following scoring method with scores ranging from 0 to 3: Score 0: normal A-lines with a continuous and regular pleural line.

Score 1: multiple separated B-lines. Score 2: coalescent B-lines pattern with alterations of the pleural line. Score 3: consolidation area and possibly a large white lung artifact. The total score was computed as the sum, which could range from 0 to 36.

## 3. Statistical Analysis

Categorical data were expressed as numbers and percentages, while continuous variables as either median and interquartile range or mean and standard deviation, according to the distribution assessed graphically and by the Shapiro–Wilk test. The presence of missing data has been reported. The study variable KL-6 was further described with an appropriate graph to analyze its distribution, and the possible association with other laboratory and clinical respiratory variables was analyzed using the Spearman correlation test. The endpoint was in-hospital IOT or mortality, assessed either from data at discharge, IOT, or death certificate. Univariable and multivariable logistic regression models were performed to evaluate the association between IOT or mortality with exposure variables. Odds ratios and 95% confidence intervals (OR—95% CI) have been calculated for all models. Logistic regression analysis was performed to evaluate the presence of risk factors for the mentioned endpoint. A multivariate model was built to include co-variates with significant interference. The *p*-value for statistical significance was set at <0.05 for all the tests. ROC curve and AUC test were calculated to assess KL-6 value with the best sensitivity, specificity, positive predicted value (PPV), and negative predicted value (NPV). All analyses were performed using statistical software STATA v16 (StataCorp. 2019. StataCorp LLC: College Station, TX, USA).

## 4. Results

In the present study, we analyzed data obtained from eighty-seven consecutive patients admitted to our hospital and that had tested positive for SARS-CoV-2 by real-time PCR analysis on rhinopharyngeal swabs. The classification of severe and critical COVID-19 patients was done based on specific parameters described in the material and methods. The clinical characteristics reported in Table 1 show that our patient population was almost equally distributed between males (52%) and females (48%). Twenty-four patients (27.6%) experienced worse outcomes, including IOT or death, whilst 63 subjects were discharged. As demonstrated in many studies since the beginning of this pandemic, also in our patient population the median age of those that were discharged was lower (67 years) compared to the patient that progressed to a critical phase (75.5 years; *p* = 0.011), confirming that age is a determining factor and positively correlates with the disease progression and exacerbation. Among the discharged patients (*n* = 63), the majority received Continuous Positive Airway Pressure (CPAP) (52.4%) or High-Flow Nasal Cannula (HFNC) (37%) support for the management of acute respiratory failure while six of them (9.5%) were treated with non-invasive ventilation (NIV). On the other side, NIV treatment has been adopted for 9 out of 24 patients (37.5%) who experienced worse outcomes. In this group of patients, LUS score was significantly higher (36 versus 29, *p* < 0.001) in comparison with discharged patients, reflecting a more extended lung involvement. Likewise, Chung CT Score was higher in patients who experienced worse outcomes (median value 15 versus 13, *p* = 0.04). Blood tests revealed a significant increase in white blood cells (WBC) and neutrophils among patients with more severe COVID-19 (*p* = 0.008 and *p* = 0.014, respectively). Furthermore, based on data that emerged from the analysis of inflammation markers, IL-6 and C-Reactive Protein (CRP) were significantly lower in survivors (25.3 pg/mL versus 115 pg/mL; *p* = 0.003 and 4.25 mg/L versus 6.5 mg/L; *p* = 0.002, respectively). When the KL-6 level was measured, we reported a higher median value (1969 U/mL) in patients who had undergone IOT or were deceased compared to discharged patients (530 U/mL; *p* < 0.001).

Therefore, to understand if KL-6 could be used as a biomarker of severity and suggest the need for intubation, we sought to perform a regression analysis. As shown in Table 2, we could demonstrate that KL-6 significantly correlated with BMI (r: 0.279, *p*: 0.009), LUS Score (r: 0.429, *p* < 0.001), and Chung Score (r: 0.390, *p* < 0.001), confirming our hypothesis.

**Figure 1:**
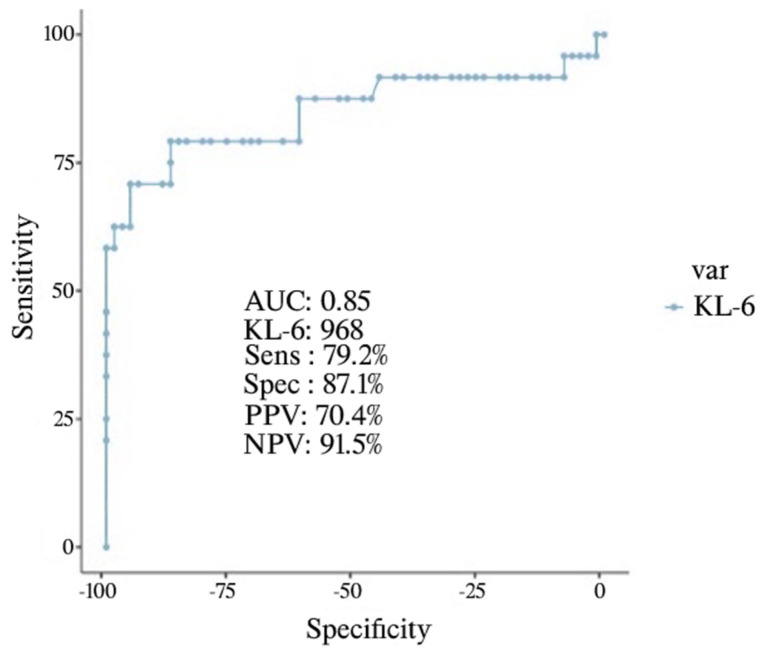
ROC Curve for KL-6 as a predictor of in-hospital mortality or IOT.

By performing multivariate analysis, KL-6 was associated with risk of death or IOT after adjusting for gender, BMI, Charlson Index, Chung Score, and PaO_2_/FIO_2_ (OR 1.003 95% CI 1.001–1.004, *p* < 0.001) (Table 3). Finally, we performed a ROC curve to define the KL-6 value with the best performance in predicting negative outcomes in patients with severe to critical COVID-19. We found that serum KL-6 value of 968 has a sensitivity of 79.2%, specificity of 87.1%, PPV 70.4%, NPV 91.5%, and AUC: O.85 for risk of death or IOT (Figure 1).

## 5. Discussion

Since the beginning of the SARS-CoV-2 pandemic, the clinical scenario related to COVID-19 has emerged as far as to be homogeneous, with an extremely broad spectrum of severity. Elevated levels of KL-6, a mucin-like glycoprotein mainly expressed by respiratory bronchiolar epithelial cells as well as type II alveolar epithelial cells, are thought to denote a disruption of alveolar epithelial cells. Indeed, abnormal values of serum KL-6 have been reported in a number of lung conditions, including acute lung injury, acute respiratory distress syndrome (ARDS), and interstitial lung diseases [22].

Although several studies have investigated multiple prognostic biomarkers in patients with COVID-19, the paucity of validated tools for predicting outcomes in these subjects still remains.

In this respect, the utility of KL-6 in COVID-19 patients has been lately explored as either a marker of severity or a prognostic biomarker. [23,24,25]. We have therefore completed a monocentric retrospective study aiming at assessing the role of KL-6 in hospitalized patients with more pronounced respiratory failure secondary to SARS-CoV-2 infection. In our study, among patients deceased or intubated, the level of serum KL-6 is significantly higher compared with patients discharged, with a median serum value of 1069 U/mL versus 530 U/mL (*p* < 0.001). According to our data, a significantly greater risk for worse outcomes should be considered for patients having a serum KL-6 value of 968 U/mL or more, with a sensitivity and specificity of 79.2% and 87.1%, respectively, and a positive predictive value of 70.4%. These findings fully match data obtained from previous studies in which elevated levels of serum KL-6 are a significant predictor of poor outcomes in patients with COVID-19 [23,24,25,26,27].

Interestingly, it has been reported that patients infected with SARS-CoV-2 with a KL-6 value persistently above 505 U/mL may be prone to develop pulmonary fibrosis, which may be irreversible when KL-6 raises above 674 U/mL [28].

Chest imaging has played a key role during the SARS-CoV-2 pandemic in detecting alterations in lung and severity quantification [29]. Severity scores have been developed for both chest computed tomography (CT) and lung ultrasound, such as Chung severity score [30] and lung ultrasound score (LUS) [31], respectively. Our study showed a significant correlation between KL-6 serum value and both Chung severity score and LUS (Table 2).

To the best of our knowledge, this is the first study showing a significant correlation between KL-6 and LUS score, highlighting the relevant role of point-of-care lung ultrasound as a ready and non-invasive tool to assess lung damage in these patients. Recent research aiming to investigate the relationship between KL-6 and chest CT scan documented a significant correlation between KL-6 and the extension of parenchymal lesions using a CT semiquantitative score. In particular, the authors found a higher frequency of crazy paving patterns and consolidations involving the right upper and middle lobe in COVID-19 patients with KL-6 > 400 U/mL [32].

Another main finding of our study was the identification of a strong correlation has been found between the serum level of KL-6 and BMI, reflecting a higher risk of lung damage in obese patients. The dramatic impact of obesity on COVID-19 severity and outcomes has been already proved [33,34]. In our study, a strong correlation has been found between the serum level of KL-6 and BMI, reflecting a higher risk of lung damage in obese patients. Accordingly, we found a significantly higher BMI in the group of patients who experienced worse outcomes, in line with existing data emerging from the literature.

A dysregulated immune system represents the hallmark of severe COVID-19. Interleukin-6 is considered a key factor in the inflammatory soup, and its inhibition has been assessed in the treatment algorithm [35]. A significant relationship between adverse clinical outcomes and abnormal levels of IL-6 has been reported [36]. IL-6 was significantly higher in patients experiencing worse outcomes compared to survivors (115 pg/mL versus 25.3, *p* = 0.003). White blood cells and neutrophils were also significantly higher in the former group compared to controls, with a mean value of 12.3 × 10^3^ cells/µL versus 8.65 × 10^3^ cells/µL (*p* = 0.008) and 11.0 × 10^3^ cells/µL versus 7.46 × 10^3^ cells/µL (*p* = 0.014), respectively (Table 1). Although the neutrophils-to-lymphocytes ratio was not found significantly different between the two groups, it shows a positive correlation with KL-6 (*p* = 0.03) (Table 2), suggesting its potential role as a surrogate marker of major risk of lung damage in these patients. The data at our disposal support the role of KL-6 as a key marker in the management of patients infected with SARS-CoV-2 in terms of severity quantification and prognosis. However, we cannot ignore that the study suffers from some limitations, such as the monocentric nature of the study and the use of retrospective data. In conclusion, our study shows that KL-6 represents a strategic tool of great utility in the diagnostic and therapeutic algorithm of more severe patients infected with SARS-CoV-2, as both a key parameter in the severity quantification of COVID-19 and a prognostic biomarker.

## 6. Conclusions

Severe and critical COVID-19 patients represent a major burden for health care systems. Prognostic stratification of patients with acute respiratory failure secondary to COVID-19 is complex because of the paucity of reliable biomarkers able to predict clinical behavior. KL-6 is a mucin-like glycoprotein mainly expressed by respiratory bronchiolar epithelial cells as well as type II alveolar epithelial cells highly expressed during COVID-19. In this study, we documented that it could be considered a valuable biomarker for predicting the risk of oro-tracheal intubation or death among hospitalized severe or critical COVID-19 patients. The relationship with imaging radiological severity score and other laboratory parameters suggests a robust association with severe prognosis among individuals during severe phases of COVID-19.

## Figures and Tables

**Table 1:** Study population characteristics.

	Discharged (*n* = 63)	Death/IOT (*n* = 24)	*p*
Age	67 (60–73)	75.5 (67–80.3)	0.011
Gender (Male)	37 (58.7)	8 (33.3)	
BMI (Kg/m^2^)	27.7 (25–31.5)	31.2 (27.7–35.2)	0.02
Charlson Index	3 (3–4)	4 (3–4)	
LUS	29 (23.8–32.3)	36 (36–36)	<0.001
Chung Score	13 (12–15)	15 (12–16)	0.04
WBC (10^3^ cell/µL)	8.65 (6.86–11.1)	12.3 (9.5–12.9)	0.008
Neutrophils (10^3^ cell/µL)	7.46 (6.31–10.2)	11 (7.66–11.5)	0.014
Lymphocytes (10^3^ cell/µL)	0.69 (0.497–1.02)	0.685 (0.44–1.07)	0.851
Eosinophils (10^3^ cell/µL)	0 (0.00–0.01)	0 (0.00–0.01)	0.859
NLR	11.8 (6.51–16.3)	13.1 (9.88–15.6)	0.113
RBC (10^6^ cell/µL)	4.72 (4.56–5.41)	4.5 (3.89–4.92)	0.003
HGB (g/dL)	13.7 (12.3–14.3)	12.6 (9.6–14)	0.058
PLT (10^3^ cell/µL)	221 (183–272)	203 (159–287)	0.382
CRP (mg/L)	4.25 (2.27–9.4)	6.5 (4–9.85)	0.002
D-Dimer (µg/L)	281(162–519)	499(435–903)	0.307
IL2R (U/mL)	1121 (809–1507)	973 (906–1737)	0.987
IL-6 (pg/mL)	25.3 (16.6–55.9)	115 (42.2–160)	0.003
KL-6 (U/mL)	530 (469–787)	1969 (1036–3669)	<0.001
PaO_2_/FiO_2_	119 (88–155)	100 (91.3–110)	0.211
Respiratory Support			
*Nasal Cannula, face mask, or non-rebreathing mask*	*4 (6.4)*	*0 (0)*	
*HFNC*	*20 (31.7)*	*2 (8.3)*	
*CPAP*	*33 (52.4)*	*13 (54.2)*	
*NIV*	*6 (9.5)*	*9 (37.5)*	

KL6 predicts negative outcomes in COVID-19 severe patients.

**Table 2:** Correlation Matrix (KL-6 Dependent Variable).

		KL6
Age	Pearson’s r	0.174
	*p*-value	0.110
BMI	Pearson’s r	0.279
	*p*-value	0.009
Charlson index	Pearson’s r	0.194
	*p*-value	0.073
LUS SCORE	Pearson’s r	0.429
	*p*-value	< 0.001
CHUNG-SCORE	Pearson’s r	0.390
	*p*-value	< 0.001
NLR	Pearson’s r	0.236
	*p*-value	0.030
D-DIMERO	Pearson’s r	0.005
	*p*-value	0.966
P/F	Pearson’s r	0.180
	*p*-value	0.101

**Table 3:** Multivariable analysis of the risk of IOT or death in severe COVID-19 patients.

		95% Confidence Interval		
	Odds Ratio	Lower	Upper	*p*
Gender	0.46967	0.101	2.18	0.334
BMI	1.09941	0.952	1.27	0.196
Charlson index	1.14269	0.631	2.07	0.66
CHUNG-Score	0.97959	0.712	1.35	0.899
PaO_2_/FiO_2_	0.99005	0.97	1.01	0.328
KL-6	1.00266	1.001	1.004	<0.001

## Data Availability

The data will be available upon request.
